# Epidemiology of a *Salmonella* Outbreak at a South African Equine Veterinary Academic Hospital Between October and December 2016

**DOI:** 10.3390/vetsci13040331

**Published:** 2026-03-29

**Authors:** Tahiyya Shaik, Henry Annandale, Daniel N. Qekwana

**Affiliations:** 1 Galley Hill Equine Surgery, Waltham Abbey EN9 2AD, UK; 2 School of Veterinary Medicine, Murdoch University, Perth, WA 6150, Australia; henry.annandale@murdoch.edu.au; 3 Faculty of Veterinary Science, University of Pretoria, Pretoria 0110, South Africa; nenene.qekwana@up.ac.za

**Keywords:** *Salmonella* outbreak, *Salmonella* Typhimurium, veterinary academic hospital, equine hospital, white rhinoceros, zoonosis, one health

## Abstract

Salmonellosis in equine medicine is of clinical and public health significance. There are no published studies on nosocomial *Salmonella* outbreaks in South Africa. Electronic medical records of patients admitted to the equine veterinary academic hospital between October and December 2016 were reviewed. The SOPs for hospital infection, prevention and control measures pre-, during, and post-outbreak, and possible zoonotic transmission were collected using structured questionnaires. *Salmonella* was isolated from 25% of patients, including a white rhinoceros. *Salmonella* Typhimurium was the most common serotype isolated from patients (42%), stables (72.2%), and clinic areas (84%). Hospitalisation duration (*p* = 0.017) and repeated faecal samples (*p* = 0.011) were significantly associated with *Salmonella* infection, but there was no association between syndromic clinical presentation of salmonellosis and *Salmonella* infection. Four students reported symptoms of salmonellosis, and one was hospitalised with a positive faecal culture. The importance of heightened biosecurity around high-risk patients cannot be over-emphasised. A continuous surveillance programme of both patients and the environment is essential to identify early lapses in infection prevention and control measures, and patient care should be optimised to reduce the length of hospitalisation. Syndromic clinical signs of salmonellosis cannot always be used to identify *Salmonella*-positive patients as previously suggested, emphasising the importance of a surveillance programme.

## Simple Summary

In this study, we investigated a *Salmonella* outbreak at a South African equine veterinary academic hospital between October and December 2016, addressing the lack of local data on the epidemiology of *Salmonella* in equine referral hospitals. The aim was to identify animal-, environmental-, and management-related risk factors associated with infection and to evaluate infection prevention and control practices. Hospital records and laboratory results of admitted patients were reviewed, and environmental sampling and questionnaires were analysed. *Salmonella* was isolated from a quarter of hospitalised patients, including a white rhinoceros, with *Salmonella* Typhimurium the predominant serotype. The number of faecal samples collected, and the duration of hospitalisation were significantly associated with infection, whereas syndromic presentation alone was not a reliable predictor of infection. *Salmonella* was frequently recovered from stables and other hospital areas, with *Salmonella* Typhimurium again predominating, indicating environmental contamination. Four students reported clinical signs consistent with salmonellosis, one of whom had a positive faecal culture, providing evidence of zoonotic transmission. The findings emphasise the need for continuous surveillance, strict biosecurity protocols, optimisation of patient management to reduce hospitalisation duration, and ongoing staff and student education to mitigate equine and zoonotic *Salmonella* transmission, thereby improving both animal and public health outcomes.

## 1. Introduction

*Salmonella enterica* is an intracellular Gram-negative, facultatively anaerobic rod-shaped bacterium of the family Enterobacteriaceae [1]. Both animals and humans are susceptible to *Salmonella* infection, and direct contact with asymptomatic carriers and infected animals is a risk factor for human salmonellosis [2,3,4,5]. *Salmonella* Enteritidis and *Salmonella* Typhimurium are most commonly reported in human infections and pose the greatest threat to public health [6]. *Salmonella* Typhimurium, *S.* Enteritidis, *S.* Antum, *S.* Heidelberg, and *S.* Newport have been reported as causes of salmonellosis in equine veterinary medicine, with the majority of cases being due to *S*. Typhimurium [3,7]. The majority of horses are asymptomatic, with less than one percent identified as being active shedders in the general equine population in previous research [8]; however, a recent study in Spain reported a higher prevalence of 25.3% in asymptomatic horses [9]. In addition, hospitalisation and stress have been shown to increase the rate of *Salmonella* shedding [1,8,10,11].

Clinical signs including pyrexia, acute diarrhoea, and leukopenia have been reported in human and equine cases, with nausea, vomiting, abdominal cramps, and constipation as additional clinical signs in humans [1,12]. Young, old, and immunocompromised patients are at a higher risk of *Salmonella* infection [1,12,13,14].

Treatment for equine salmonellosis includes intravenous fluids, plasma therapy, colloid therapy, nonsteroidal anti-inflammatories, antidiarrheal preparations, cryotherapy, and heparin. Antimicrobial use is discouraged as the disease is self-limiting [15,16,17]. However, if there is evidence of extra-intestinal infection, of chronic recurrent enteric disease, or in foals younger than 90 days, antimicrobial use is considered [17].

Increased environmental burden and poor hygienic conditions remain the main sources of infection for immunocompromised patients [7,9,18]. Studies have also reported nosocomial *Salmonella* outbreaks in equine veterinary teaching hospitals that in some cases resulted in hospital closure [10,11,19,20,21,22]. Therefore, host susceptibility and environmental persistence remain the main factors contributing to *Salmonella* outbreaks in veterinary hospitals [1,11]. Inadequate infection prevention and control measures may also be contributing to the spread of infection and the occurrence of outbreaks [1,5,9].

Although *Salmonella* infection and outbreaks have been well documented in other countries, there are no published studies on the prevalence and occurrence of *Salmonella* outbreaks in equine veterinary hospitals in South Africa. Therefore, the objective of this study was to investigate the prevalence and animal, environmental and managerial factors associated with the 2016 *Salmonella* outbreak at an equine veterinary academic hospital. In addition, we aimed to investigate the zoonotic potential associated with the outbreak.

## 2. Materials and Methods

### 2.1. Data Source

An electronic dataset of the medical records of patients admitted to the equine veterinary academic hospital between 30 October 2016 and 12 December 2016 was reviewed as routine faecal sampling and *Salmonella* culture identified an increase in the number of *Salmonella*-positive patients during this period. The *Salmonella* status of the patients five days prior to the designated period was also reviewed as a baseline. All patient information obtained was part of routine care. However, on patient admission, clients signed a consent form allowing patient data to be used for further research. In addition, consent for the use of the data was obtained from the hospital director. The following variables were obtained for each patient: signalment, presenting complaint, diagnosis, outcome, location of stable, duration of hospitalisation, clinical examination, and laboratory results.

The results of the environmental samples were obtained from the bacteriology laboratory and reviewed for the designated period (30 October 2016 to 12 December 2016). Environmental culture results a month before the suspected outbreak were also included to determine a baseline. The following information was obtained concerning each sample: date, culture, serotyping, and the location in the hospital. For the purpose of this study, environmental samples were divided into clinic areas and stables. The hospital has a total of 52 stables, divided into six different rows to separate highly infectious patients from the young or clinically healthy. The mares and foals admitted are stabled in the paediatric row whereas patients with gastrointestinal disease are stabled in the colic row. Clinically healthy patients that are admitted for routine procedures are stabled in the general row. Infectious patients are stabled in either semi-isolation or in isolation, depending on the risk of transmission to other patients. The padded downer stable, which is situated separately from the other stables, is used for patients that are recumbent or that are at a high risk of recumbency. All stables and clinical areas have adequate drainage. A diagram of the stables can be found in the Supplementary Information.

### 2.2. Patient Faecal Samples

The standard operating procedure (SOP) of the equine hospital is that faecal samples for *Salmonella* culture are taken from every equine patient on admission, including outpatients. Outpatients are defined as patients presented to the equine hospital for examination, diagnostic procedures, or treatment who are not admitted for hospitalisation and are discharged on the same day. In this study, faecal samples were collected from freshly voided faeces within 2 h of defaecation, and no rectal collection of faeces was performed unless it was part of the diagnostic work-up of the case. If no faeces could be collected on admission, the first faecal sample would be submitted as soon as faeces were obtained. Faecal samples were also collected from hospitalised patients every Monday and Thursday and on patient discharge. The number of faecal samples depends on the duration of hospitalisation. The samples were collected in sample pots and refrigerated before they were collected by the bacteriology laboratory for processing.

### 2.3. Environmental Samples

Clinic area swabs were collected once a year in February, including from spaces such as offices, corridors, storages areas, theatres, work-up areas, and drug stores. The layout of the equine clinic, traffic patterns for patients and personnel, and the use of the spaces in the clinic were considered when determining sample sites. Hand- and foot-contact areas were sampled individually using separate cloths to avoid cross-contamination between sites. Sampling intensity was higher in high-risk than low-risk areas and included five random swabs once a month. The SOP required that a stable not be reused until the *Salmonella* culture results of the previous patient were received and the stable was cleaned appropriately. Different cleaning SOPs were in place depending on the *Salmonella* status of the patient. For a *Salmonella*-positive patient, the organic material was collected in black bags for incineration, the type of cleaning agents was changed, and the contact time of those agents was increased (details provided in the Supplementary Information). The wall and floor of the stable of a *Salmonella*-positive patient were swabbed after cleaning to ensure a negative status. Positive stable swabs prompted cleaning and repeated sampling until a negative status was confirmed.

Samples were collected using a commercial floor mop (ADDIS^®^ Johannesburg, South Africa) similar to the Swiffer Sweeper^®^ Charleston, WV, USA) and dry new household cleaning cloths. A single cloth was used at each site, with approximately 80% of the surface being swabbed. For floor and wall samples, a cloth was attached to the floor mop to enable sampling. Between swabbing each site, the floor mop was disinfected with 70% ethanol and left to dry. A cloth held in a gloved hand was used to swab hand-contact areas, with gloves being changed between each sample. After sampling, each cloth was placed in a labelled sterile plastic bag. These samples were sent to the bacteriology laboratory for *Salmonella* isolation. If swabs were positive, subsequent testing was performed after cleaning until the test was negative.

### 2.4. Bacterial Isolation

Samples were cultured in the laboratory via the enrichment method. One gram of faeces was added to nine millilitres (mL) of buffer peptone water and incubated for 24 h at 37 °C. Environmental swabs were added to 90 mL of buffer peptone water, with the time and temperature of incubation remaining the same. One millilitre of the solution was added to a 10 mL tetrathionate broth and incubated for a further 24 h at 42 °C. This solution was plated onto Xylose Lysine Deoxycholate (XLD) agar and incubated for 24 h at 37 °C. The growth of black colonies indicated that it was a *Salmonella* species. The suspected colonies were then plated onto XLD and blood agar and incubated for 24 h at 37 °C. The colonies that did swarm were further subjected to a biochemical test, named the Analytical Profile Index (API) 10S (manufactured by bioMérieux^®^ Marcy-l’Étoile, France), which is a miniaturised biochemical identification system containing 10 metabolic tests used to identify Gram-negative bacteria, including *Salmonella*, based on their biochemical reaction profile [23]. Pure *Salmonella* cultures were serotyped at the Onderstepoort Veterinary Institute following the methods described by the White–Kauffmann–Le minor scheme [24,25]. Isolates that could not be completely serotyped were classified as Salm Poly OMD, while isolates partially identified were classified as Salm II.

### 2.5. Case Definition

Previous studies have used syndromic clinical signs to identify a suspected salmonellosis case and defined this as the presence of two of the following clinical signs: pyrexia, neutropaenia, or diarrhoea, in the absence of another apparent cause [1,18,26]. In this study, we defined a clinical *Salmonella* case based on the presence of at least two of the syndromic clinical signs on admission, with a confirmed positive faecal culture. A carrier was defined as a clinically healthy patient with a positive *Salmonella* faecal culture within 24 h of admission. Hospital-acquired-infection was defined as those that had an initial negative *Salmonella* faecal culture but a positive culture on a faecal sample collected more than 24 h after admission.

### 2.6. Infection Prevention and Control

Information regarding infection prevention and control, before, during, and after the outbreak, was collected using a questionnaire. The questionnaire was completed by the equine clinic stable manager in charge of infection prevention and control at the equine hospital. The information collected related to cleaning procedures, infection prevention and control measures, environmental swabbing procedures, patient monitoring, and any changes that occurred before, during, and after the outbreak. The SOPs and cleaning protocols of the equine clinic were also reviewed.

### 2.7. Students’ Exposure to the Salmonella Outbreak

A structured questionnaire was used to collect information relating to *Salmonella* infection from veterinary students present at the equine veterinary academic hospital during the time of the outbreak. This questionnaire was pre-tested, validated, and approved by the Human Ethics Committee at the University of Pretoria (Ethics Reference No: 83/2018). Retrospective ethical approval was obtained as the conceptualization of the study occurred after the outbreak. Written consent was obtained from each participant, and the participants were informed of the objective of the study. In addition, consent was sought from the students who received medical attention.

### 2.8. Data Management and Analysis

The data was checked for inconsistencies and missing documentation; none were noted. For the purposes of the analysis the variables diagnosis, age, and breed were classified into categories. Clinical diagnosis was classified into six categories depending on the body system involved, namely, head, abdomen, reproductive, limbs, systemic infection, and healthy. Age was classified into three categories: foal (<1 year), adult (1–18 years), and senior (>18 years). Breed was classified into three categories: European, American, and African. The European category consisted of Warmblood, Thoroughbred, Miniature donkey, Friesian, Flemish horse, Arabian, and Miniature horse; the American category of American saddler and Appaloosa; and the African category of Nooitgedacht, White rhinoceros, and Boerperd.

Data analysis was performed using IBM SPSS Statistics software 31.0.2.0©. The Shapiro–Wilk test was used to test the normality of continuous variables. Age and length of stay were not normally distributed; therefore, median and interquartile range were reported. Proportions and 95% confidence intervals were computed for all categorical variables in the dataset. Association between the proportions of positive cases and categorical variables (diagnosis, clinical outcome, location, pyrexia status, diarrhoea status, neutropaenia status, and carrier status) was assessed using Chi Squared test or Fisher’s exact test where >20% of the cells had expected frequencies ≤ 5. The variables of diagnosis and location had too many categories to include in the univariable model in their original form and therefore were re-coded.

A univariable logistic regression model was fit to assess the relationships between breed, pyrexia, diarrhoea, neutropaenia, clinic service, diagnosis, duration of stay, and number of faecal samples and the outcome *Salmonella* status. The significance of the results was set at α ≤ 0.05. Odds ratios (ORs) and their corresponding 95% confidence intervals were calculated for all predictor variables. Due to the relatively small number of cases available for analysis in relation to the number of potential predictor variables, a multivariable logistic regression model was not performed to avoid model overfitting and unstable coefficient estimates. Correlation between categoric variables was assessed using polychoric correlation tests. Pearson’s correlation test was used to assess the correlation between continuous variables. Correlations were used to summarise relationships between variables in a descriptive manner. Accordingly, *p*-values were not computed.

## 3. Results

### 3.1. Clinical Cases

#### 3.1.1. Demographic Profile of Patients

A total of 48 animals were hospitalised during the study period, with a median stay of five days per animal (Q1:0; Q3:58). The majority of cases were equine, except for one white rhinoceros (*Ceratotherium simum*). The median age of hospitalised animals was 6.2 years (Q1:0.08; Q3:30.75). Most hospitalised animals were adults (69%), and the majority of animals were European breeds (69%), followed by African breeds (23%). Most clinical cases involved the limbs (33%), followed by the abdomen (31%). Almost half of the patients were stabled in the general row (44%), with a few animals (2%) in the isolation row. The median number of faecal samples was two per animal (Q1:0; Q3:17). Of the hospitalised patients, 25% had pyrexia, 12% had diarrhoea, and 21% were neutropaenic (Table 1).

#### 3.1.2. Distribution of *Salmonella* Cases

**Figure 1:**
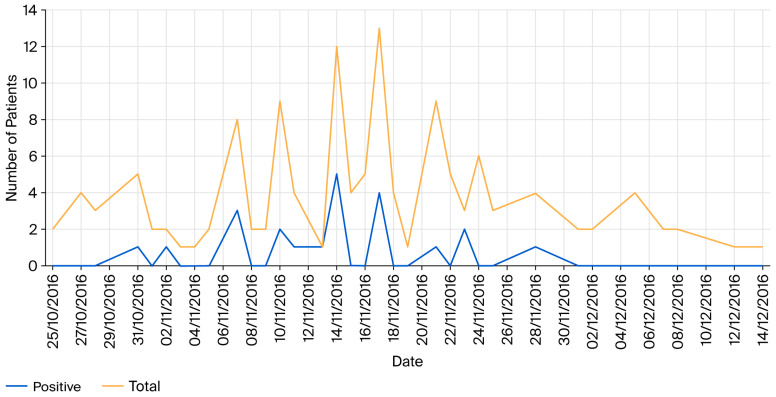
Distribution of patients tested and *Salmonella*-positive between October 2016 and December 2016.

*Salmonella* was isolated from 25% of hospitalised animals during the study period, including the white rhinoceros (*C. simum*). The first positive case, the white rhinoceros, was identified on 31st October. An increase in the number of *Salmonella*-positive patients was noted on 7th November, with a peak on 14th November (Figure 1). The median age of the *Salmonella*-positive patients was 9.8 years (Q1:0.08; Q3:18). The median length of stay at the hospital for all positive cases was nine days (Q1:5; Q3:58), with the median number of faecal samples being four (Q1:1, Q3:17). *Salmonella* infection was more common in female (19%) compared to male patients (6%). The European breed (19%) exhibited the highest proportion of positive cases. *Salmonella* was more common in adult (19%) patients, followed by foals (4%) and seniors (2%). In addition, *Salmonella* was isolated from a higher proportion of cases on the medicine service (19%) compared to the surgery service. *Salmonella* was also isolated from more abdominal (15%) cases, followed by reproduction (6%) cases. The majority of the *Salmonella*-positive patients were discharged (23%), with only one dying (2%) and none being euthanised. The majority of positive *Salmonella* cases were *Salmonella* hospital-acquired infections (58%), followed by carrier patients (25%) and clinical *Salmonella* patients (17%).

*Salmonella* was isolated in high proportions from patients without diarrhoea (19%) and pyrexia (15%) compared to those with diarrhoea (6%) and pyrexia (10%). Based on the syndromic clinical definition of salmonellosis, 17% (8/48) of all hospitalised cases had at least two of the clinical signs of a suspected *Salmonella*-positive case. Of the positive *Salmonella* patients, less than 50% (5/12) had at least two syndromic clinical signs of salmonellosis, and of the negative *Salmonella* patients, almost 10% (3/36) had at least two of these signs (Table 1).

#### 3.1.3. Univariable Association

There was no significant association between pyrexia (OR 2.96, CI 0.72–12.17; *p* = 0.13), diarrhoea (OR 3.67, CI 0.63–21.35; *p* = 0.15), neutropaenia (OR 1.5, CI 0.3–7.53; *p* = 0.62) and *Salmonella* status. However, significant associations between clinic service (OR 4.17, CI 1.09–20.47; *p* = 0.038), diagnosis (OR 7, CI 1.67–29.38; *p* = 0.008), duration of stay (OR 1.26, CI 1.04–1.52; *p* = 0.017), and number of faecal samples (OR 1.70, CI 1.13–2.57; *p* = 0.011) and *Salmonella* status were observed.

#### 3.1.4. Polychoric Correlation

Clinical service was found to be moderately correlated with the presence of neutropaenia (r = 0.6434) and strongly correlated with the presence of pyrexia (r = 0.8003), while neutropaenia was moderately correlated with the presence of diarrhoea (r = 0.4012) and pyrexia (r = 0.5175). The presence of pyrexia was moderately correlated with the presence of diarrhoea (r = 0.6275). Pearson’s correlation test identified a strong correlation between the number of faecal samples taken and the duration of stay at the hospital (r = 0.96210).

#### 3.1.5. *Salmonella* Typing

*Salmonella* Typhimurium was the most common serotype isolated amongst the positive *Salmonella* patients (42%), followed by *Salmonella* Virchow (17%) and *Salmonella* Poly OMD (17%). Only one isolate of *Salmonella* Braenderup, *Salmonella* Heidelberg, and *Salmonella* II was obtained in this study (8% each) (Table 2).

### 3.2. Environmental Sampling

#### 3.2.1. Distribution

**Figure 2:**
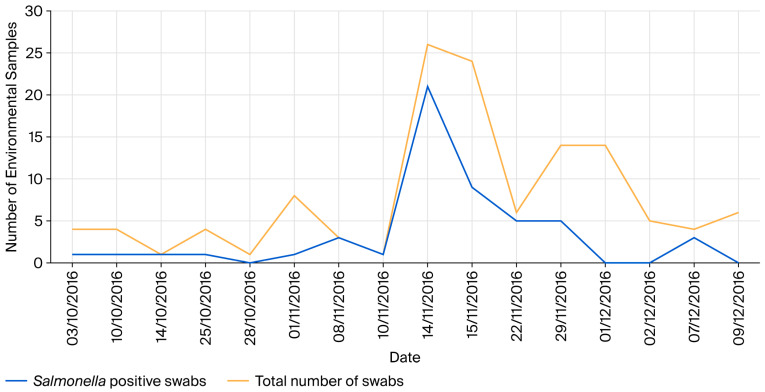
Distribution of positive *Salmonella* environmental samples at the Onderstepoort Veterinary Academic Hospital between 31 October 2016 and 11 December 2016.

One month prior to the designated period (30 October 2016 and 12 December 2016), up to four environmental samples were tested from the stables of positive *Salmonella* patients, with a maximum of one positive sample each time. On 1st November, the number of environmental samples tested increased as the five random monthly high-risk area swabs were sampled, but the number of positive cultures remained the same. Another increase in the number of environmental samples tested was observed between 10th November and 15th November, with a significant increase in the number of positive samples during the same period (Figure 2). This increase in environmental sampling was prompted by an increase in the number of *Salmonella*-positive patients.

#### 3.2.2. Stables

Of the 24 stables swabbed, 16 (67%) were *Salmonella*-positive and all 16 samples were serotyped. Four of the initial positive stables tested positive again after cleaning but only two of these samples were serotyped and were identified as *S.* Typhimurium. Of all the isolates serotyped, 72% were typed as *S.* Typhimurium and 16% were *Salmonella* Poly OMD. Only one isolate was typed as *Salmonella* II (6%) and another as *Salmonella* Schwarzengrund (6%) (Table 2).

#### 3.2.3. Clinic Areas

Twenty-five clinic areas out of the fifty-four tested at the equine veterinary academic hospital were positive for *Salmonella*. Of the twenty-five positive areas, only two tested positive again after cleaning, both being in the semi-isolation row. These two repeat positive cultures were not serotyped. *S*. Typhimurium (84%) was most common, followed by *S*. Newport (8%). One serotype of *S*. Heidelberg (4%) and one of rough-untypeable *Salmonella* (4%) were also identified (Table 2).

### 3.3. Students

Of the 55 students that completed their equine rotation between the 30 October 2016 and the 12 December 2016, 45 (82%) agreed to participate in the study. Four (9%) had symptoms of salmonellosis either during their clinical rotation or immediately afterwards. Of these students, 25% (1/4) had pyrexia and consulted a doctor, and 50% (2/4) had nausea, vomiting, abdominal cramps, and diarrhoea. One student was admitted to hospital and on faecal culture was confirmed positive for *Salmonella*. Unfortunately, sample serotyping was not performed.

### 3.4. Source of the Outbreak: White Rhinoceros (Ceratotherium simum)

Based on the temporal patterns, the results in this study suggest the point source for the outbreak was the white rhinoceros. Moreover, the white rhinoceros was one of the first patients admitted before the outbreak was identified. The two-month-old orphaned female rhinoceros was referred to the equine clinic on the night of the 30 October 2016 for colic of two-day duration, which had not improved despite treatment by the local veterinarian. She was born in the wild and orphaned at one month of age as a result of poaching, after which she was rescued and cared for at a rhinoceros orphanage. On presentation, she was admitted to a semi-isolation stable, and a clinical examination, blood tests, and abdominal radiographs were performed. A diagnosis of colic due to sand impaction was made, and due to the duration of the colic and deteriorating clinical state, surgical correction was the best treatment option. In addition to the sand impaction, a large colon displacement was diagnosed during exploratory laparotomy. This was manually corrected, and an enterotomy was performed. She developed a purulent nasal discharge, dyspnoea, and diarrhoea between 24 and 48 h post-operatively. The rhinoceros was severely neutropaenic pre-operatively, which progressively worsened after surgery. Her clinical state progressively deteriorated, with the diarrhoea and dyspnoea worsening daily, and she died from respiratory arrest on the 4 November 2016. The post-mortem revealed extensive ulcerative colitis exacerbated by Klebsiella septicaemia. By case definition, this case was classified as a *Salmonella* carrier patient as she tested positive for *Salmonella* within 24 h of admission. The serotype isolated from this case was *Salmonella* Typhimurium, and the only two clinic areas that tested positive after cleaning (*S*. Thyphimurium serotype) were in the semi-isolation row where the rhinoceros was stabled. In addition, the hospitalised *Salmonella*-positive student solely oversaw the white rhinoceros and no other patient with nosocomial infections. Due to the sensitive nature of this case, only essential personnel were allowed to enter semi-isolation; however, this was difficult to control at night when there were limited staff and students monitoring the patients.

### 3.5. Infection Prevention and Control

The results of the biosecurity questionnaire were as follows.

#### 3.5.1. Pre-Outbreak

The protocol prior to the outbreak was a yearly swab of all areas in the clinic. This was duly performed in February 2016, eight months prior to the outbreak. The cleaning was performed according to an SOP. The disinfectants used included general detergent, bleach, and quaternary ammonium compound (QAC) products. Foot baths containing peroxygen were present at every exit and entrance to the clinic, high-risk rows, and outside high-risk patients’ stables.

#### 3.5.2. During the Outbreak

Fifty-three percent (41/78) of the environmental samples tested positive for *Salmonella*. This confirmed the outbreak status. Once the increase in positive samples was noted, the hospital was closed to new admissions, current patients were discharged as soon as possible, and the cleaning regime was changed to the SOP for a *Salmonella* outbreak. The same disinfectants were used as in routine SOP, but the frequency of cleaning increased and the concentration of the QAC product increased by 2.5 times. The clinic was closed to provide an optimal cleaning environment. Of the initial positive environmental samples, only 15% (6/41) tested positive after cleaning. These areas were easily isolated from the rest of the hospital and were cleaned and retested until a negative result was obtained. This allowed for the reopening of the clinic. The equine clinic was closed for a period of six weeks.

#### 3.5.3. Post-Outbreak

After the outbreak, the protocol for surveillance was changed to five random area swabs once a month and a yearly swab of all clinic areas at the end of the year. This has subsequently been changed to biannual swabs of all the clinic areas with five random swabs monthly, which includes all clinic areas and not only high-risk areas as before. However, no changes have been made to the cleaning SOP or the detergents used. The only modification was the chemical used in the footbaths. This was changed from peroxygen to a QAC product.

## 4. Discussion

The proportion of *Salmonella*-positive patients during this outbreak (25%) was higher compared to a 7% prevalence in patients hospitalised at the equine clinic between January and September 2016. Dallap Schaer et al. [20] reported a lower proportion (3.8%) of *Salmonella* among equine hospitalised patients in the USA. In the same country, Cummings et al. [21] and Schott et al. [19] also reported lower proportions of *Salmonella* infections among equine patients: 0.8% and 13%, respectively. However, our findings were similar to a 25% prevalence of infection reported in one study [27] and a 22% reported in another study [18] among equine patients in the USA. A more recent study in Spain also identified a prevalence of 25% [9]. The higher proportion of infected patients observed in this study, compared with previous reports, may reflect suboptimal implementation of infection prevention and control measures within the hospital—such as insufficient cleaning and disinfection of stables and clinic areas—or alternatively, enhanced sensitivity due to repeated faecal sampling, as suggested in the Spanish study [9]. Additionally, the proportion of observed cases in this study could be due to the level of *Salmonella* carriers in the South African equine population. Therefore, compliance with infection prevention and control principles must be enforced and monitored, and the rigorous screening of all patients admitted to the equine hospital is essential.

Of the *Salmonella*-positive patients, only 17% were classified as clinical *Salmonella* cases based on clinical presentation at admission. In contrast, Dallap et al. [20] and Ward et al. [22] in the USA reported *Salmonella* clinical signs in all *Salmonella*-positive cases in their studies. Cummings et al. [21] in the USA described clinical signs in 50% of *Salmonella*-positive patients. The reasons for the dissimilarity could be due to differences in study populations. For example, Dallap et al. [20] and Ward et al. [22] reported salmonellosis in clinical patients, whereas our study looks at clinical and non-clinical patients.

We observed no significant association between *Salmonella* status and the syndromic clinical signs of salmonellosis. This is in agreement with the findings of Alinovi et al. [10] and Burgess et al. [18]. In contrast to other studies [28,29], our results suggest that syndromic surveillance may not be effective in identifying suspected *Salmonella* cases. This also highlights the importance of *Salmonella* carrier patients in a hospital environment. Routine faecal cultures should be performed for hospitalised patients, and strict infection prevention and control measures must be instituted throughout the hospital to prevent new cases and potential spread of the infection throughout the hospital [9,26,30].

*Salmonella* Typhimurium was the most common serotype isolated among patients in the study population. This is not unique as this serotype has previously been isolated in high proportions in healthy and clinical equine cases [31,32], including horses at necropsy [33]. In addition, this serotype has been reported as a cause of outbreaks in a number of studies including those by Ward et al. [22] at Purdue University in 2000 and Schott et al. [19] at Michigan State University in 1996.

The internal medicine unit had a higher proportion of *Salmonella* cases; patients admitted there had approximately four times higher odds of testing positive for *Salmonella* than those in the surgery unit. This is unsurprising as *Salmonella* infection in this study was higher in abdominal cases, which are handled by the internal medicine team. Sevenfold higher odds of a *Salmonella*-positive faecal culture were observed in horses in the abdominal category. The presence of gastrointestinal disease, especially colic, has also been identified as a risk factor for *Salmonella* infection in horses [10,14,18]. In addition, internal medicine patients are more likely to be critically ill and immunocompromised. Biosecurity around these patients should be increased, and extra precautionary infection control measures must be taken when managing patients with gastrointestinal diseases to prevent the spread of *Salmonella* in the hospital.

The observed strong correlation between the number of faecal samples collected and the duration of hospitalisation was expected, as prolonged inpatient stays inherently allow for the collection of a greater number of faecal samples. In the univariable association, increased duration of stay in hospital was also associated with a *Salmonella*-positive faecal culture. For one unit increase in the duration of stay it was expected, on average, a 1.26 times higher probability of *Salmonella* positive status. Kim et al. [28] also found in a USA-based study that equine patients hospitalised for a longer time had an increased probability of testing positive for *Salmonella*. Similarly, Ward et al. [11] reported that a longer duration of stay in hospital was associated with an increased proportion of *Salmonella*-positive horses in another USA study. The increased risk of infection could be due to increased exposure to the contaminated environment or infected patients. It is also possible that a longer stay at the hospital increases the stress level of patients, making them more susceptible to infection or increasing *Salmonella* shedding. Optimising patient care and treatment to improve patient prognosis and shorten the length of stay could reduce the risk of *Salmonella* infection. While this finding may partly reflect increased sampling frequency, the surveillance program had been in place prior to the outbreak, suggesting that additional factors may also have contributed to the observed increase in *Salmonella*-positive cases. In addition, infection prevention and control measures need to be implemented and monitored around critical patients [29]. Where possible, non-critical patients must be housed in an area further away from critical patients and not be hospitalised for longer than necessary.

The number of faecal samples was also associated with an increased likelihood of detecting *Salmonella*-positive patients. Each one-unit increase in faecal samples was associated with a 70% increase in the odds of *Salmonella*-positive status. This finding may reflect the structured sampling protocol, whereby patients that remain hospitalised for longer periods are sampled more frequently, increasing the likelihood of *Salmonella* detection. In support of our findings, Van Duijkeren et al. [34] in the Netherlands reported that by repeated faecal sampling from each patient, there was an increase in the proportion of positive animals from 64% to 86%. It is generally accepted that three to five faecal samples are required for accurate identification of *Salmonella* [35,36]. In addition, the median number of faecal samples collected in this study was four, consistent with a recent Spanish study that reported *Salmonella*-positive results only on the fourth or fifth sample [9]. These findings support current recommendations that repeated faecal sampling over the course of hospitalisation improves the likelihood of identifying *Salmonella*-positive patients [1]. Previous studies have suggested daily faecal sampling to increase the sensitivity of *Salmonella* testing [9,29]; however, the cost vs. the benefit of this needs to be carefully considered.

Only one *Salmonella*-positive patient, the white rhinoceros (*C. simum*), died in this study. The low mortality rate observed could be due to the low virulence of the *Salmonella* serotype identified. It is also possible that the immune system of the patients could have played a role. Adults typically possess a more mature immune system compared to younger individuals; the white rhinoceros in this case was only two months of age. In addition, reports have also identified that white rhinoceroses exposed to stressful conditions may develop *Salmonella* enterocolitis, which can be fatal [37]. Further epidemiological studies are needed to better understand the disease in white rhinoceros (*C. simum*) populations.

The presence of a white rhinoceros draws attention to another interesting aspect of this study—the presence of wildlife in an equine hospital. Recent studies have highlighted the role of wildlife in the epidemiology of *Salmonella* and the associated public health risks [38,39]. As wildlife medicine develops, so does the need for intensive care facilities. It is even more critical to provide this care for endangered species, such as the rhinoceros. When a wildlife case presents, due to the unique nature and the lack of information, different services such as medicine, surgery, anaesthesiology, and radiology are used, thus leading to widespread involvement within the hospital as well as the potential spread of pathogens throughout the hospital. The white rhinoceros in this study tested positive for *Salmonella* within 24 h of admission. Unlike horses, it is not known if rhinoceroses can be asymptomatic carries and shedders of *Salmonella* [40]. Factors that affect *Salmonella* shedding in rhinoceros have not been identified; however, capture and prolonged transport have been reported to alter the faecal microbiota composition of the white rhinoceros, potentially increasing the risk of infectious intestinal disorders [40]. In addition, the white rhinoceros admitted to the equine clinic was subject to all risk factors for increased shedding of *Salmonella* in horses. These included long transport distance, the administration of antibiotics, colic, gastrointestinal surgery, diarrhoea, and nasogastric intubation [1,10,18]. This patient also developed all the syndromic clinical signs of salmonellosis. Therefore, efforts must be made to reduce stress during the capture, transportation, and clinical care of captive wildlife patients to reduce the risk of infectious intestinal disease and shedding of *Salmonella*. Nonetheless, this study highlights the need for more research on the shedding of *Salmonella* in the white rhinoceros.

Environmental contamination with *Salmonella* was higher than the 0.5% prevalence reported at Cornell University [21], 12% prevalence at Michigan State University [19], and 4% prevalence at Colorado State University [27]. A lower (1.1%) proportion of environmental *Salmonella*-positive samples were also reported at Purdue University in 2005 [22]. The high prevalence of environmental *Salmonella*, with the most predominant serotype the same as that of the patients (*S.* Typhimurium), is suggestive of a lapse in infection prevention and control measures [41]. Moreover, environmental surveillance swabs were only carried out once a year before the outbreak. Although patients were screened for *Salmonella* upon admission, culture results required several days to become available. During this period, infected patients may intermittently shed the organism and contaminate the environment before a positive diagnosis is confirmed, supporting the need for broader environmental surveillance. We propose a new protocol for sampling the entire hospital every three months and for the monthly swabs to be continued. Due to financial limitations, the hospital has subsequently instituted biannual environmental surveillance swabs with a continuation of the five random swabs collected monthly [42]. The economic impact that environmental surveillance imposes on a veterinary hospital cannot be overlooked, but further work is needed to truly understand the financial implications [43].

Adequate infection prevention and control measures are essential to help prevent or at least minimise the spread of infection and decrease the risk of an outbreak [7,9,26,41]. In addition, environmental surveillance would aid in the identification of environmental reservoirs and the early detection of environmental contamination to prevent nosocomial infections [43,44]. Environmental and hygiene practices and the control of traffic through the hospital, especially in high-risk areas, can also minimise the spread of *Salmonella* [7,13,19,27,45], while environmental *Salmonella* surveillance can be used to identify high-risk areas and monitor the effectiveness of the cleaning programme [5,7,41]. The above measures are even more vital in an academic hospital where there is a high number of students passing through regularly. When a wildlife case is admitted, these measures need to be tightened, as cases of this nature attract visitors, thus resulting in the increased risk of pathogens being spread throughout the hospital. In addition, these cases should be appropriately isolated to minimise both traffic around and stress on the animal. It is important to note that these measures are already implemented at this equine veterinary academic hospital; however, it is possible that they may not have been adequately enforced. The closure of this equine hospital was shorter compared to other studies, suggesting that the cleaning and disinfection programme implemented for an outbreak was effective as well as implying the rapid detection of the outbreak status. This is in contrast with studies by Ward et al. [22] and Tillotson et al. [27] in the US, which reported hospital closures of three months during *Salmonella* outbreaks at teaching hospitals.

The changes made to the cleaning SOP during the outbreak compared to pre-outbreak consisted in an increase in the concentration of the disinfectant and an increase in the frequency of cleaning. Jang at al. [46] found that 0.3% sodium dichloroisocyanurate (NaDCC) significantly reduced bacterial load with a contact time of one minute, whereas 0.02% QACs combined with 0.03% citric acid (CA) required > 5 min and 0.5% potassium peroxymonosulfate (MPS) > 10 min. The main post-outbreak modification involved replacing peroxygen-based disinfectants in footbaths with QAC. Another study by Horning et al. [47] in the USA reported that both peroxygen and QAC disinfectants could be used to decrease bacteria on the soles of overboots but the amount of reduction was modest. Footmats and footbaths are mere deterrents of unnecessary traffic, and their impact on pathogen load reduction is limited regardless of the disinfectant used, suggesting that contact time and concentration must be appropriate for the disinfectant used [47,48]. They can help reduce the mechanical transfer of contaminated material, but their effectiveness depends on being properly maintained and kept free of organic debris as this can potentially inactivate the disinfectant [48,49].

Although *Salmonella* species are zoonotic, to our knowledge, this study and that of Schott et al. [19] from Michigan State University are the only studies that have reported potential zoonotic salmonellosis among students during an outbreak at an equine veterinary teaching hospital. In this study, four students reported salmonellosis symptoms, with one being hospitalised and diagnosed with salmonellosis. It is possible that the number of infected students could be higher due to under-reporting [2,19]. Nonetheless, the zoonotic potential of *Salmonella* infection during an outbreak cannot be over-emphasised. Therefore, staff and students should be educated on infection prevention and control measures to ensure that these measures are implemented successfully. This would include understanding *Salmonella* transmission, risk-based patient triage, and isolation protocols, the need for personal protective equipment and hand-hygiene protocols, environmental decontamination, movement control within the hospital, and ongoing surveillance systems [50,51]. This training should be performed at the start of the students’ equine rotation and yearly for staff. Furthermore, during a *Salmonella* outbreak investigation, including staff and students will allow the true extent of the outbreak to be established.

The most important finding of this study demonstrates that patients, the environment and students are intrinsically interconnected and cannot be considered in isolation, highlighting the need for a One Health approach to the management of *Salmonella* in an academic equine veterinary hospital.

The results of this study should be interpreted in the light of a few limitations. Although the veterinary teaching hospital is the only academic hospital in South Africa, it is possible that other equine veterinary hospitals might differ in how patient care, infection prevention and control, and *Salmonella* case identification are implemented. The retrospective design of the study may introduce recall bias among personnel and students. The small sample size used in this study could have also resulted in the overestimation of the magnitude of association between the outcome and predictor variables. In addition, interpretation of some associations should be cautious, as wide confidence intervals indicate limited precision. Although multivariable analysis is generally considered more robust, it was not feasible due to the small sample size. Patient information on previous antimicrobial use was not available to the researchers, and this could have resulted in the overestimation of the overall prevalence of *Salmonella*-positive cases in this study. Moreover, antibiotic exposure has been reported as a risk factor for *Salmonella*-positive cases in equine medicine. It is also possible that the prevalence of *Salmonella*-positive students could have been higher than reported due to the fact the disease in humans can be self-limiting and students may have been uncomfortable in disclosing possible gastrointestinal disease. In addition, due to privacy concerns, the required ethics approval for a questionnaire survey of staff could not be obtained. The exact concentration of the disinfectant used as well as the exact contact time was not obtained. The lack of molecular typing of isolates cultured from a positive student, the environment, and patients makes it difficult to conclusively state the role of the environment, patients, and students in the transmission of *Salmonella* species during the outbreak.

Nonetheless, the results of this study provide the basis for future studies on the role of the environment, patients, and students in the epidemiology of *Salmonella* in equine veterinary hospitals.

## 5. Conclusions

*Salmonella* Typhimurium was the most common serotype identified during the outbreak, with a white rhinoceros identified as the source. Further research needs to be carried out to investigate the role of wildlife in the epidemiology of *Salmonella* and to investigate the shedding of *Salmonella* in rhinoceros. The importance of a One Health approach in managing *Salmonella* infections in academic equine veterinary hospitals cannot be overstated. Biosecurity should be increased around high-risk patients and a continuous surveillance programme of both patients and the environment must be implemented to identify an early lapse in infection prevention and control measures. In addition, patient care and treatment should be optimised to reduce the length of stay in hospital. Educating both staff and students on infection prevention and control measures will help ensure that these measures are implemented successfully. Syndromic clinical signs of salmonellosis cannot always be used to identify *Salmonella*-positive patients as previously suggested, emphasising the importance of a surveillance programme.

## Figures and Tables

**Table 1:** Distribution of patients and *Salmonella*-positive patients hospitalised at the equine veterinary academic hospital between 30 October 2016 and 12 December 2016.

	Patients			*Salmonella*-Positive Patients		
	Number	%	95% CI	Number	%	95% CI
Sex						
Male	24	50	36–64	3	6	1–13
Female	24	50	36–64	9	19	8–30
Species						
Equine	47	98	94–102	11	23	11–35
Rhinoceros	1	2	0–6	1	2	0–6
Breed						
European breeds	33	69	56–82	9	19	8–30
African breeds	11	23	11–35	1	2	0–6
American breeds	4	8	1–16	2	4	0–16
Age						
Foal	9	19	8–30	2	4	0–10
Adult	33	69	56–82	9	19	8–30
Senior	6	12	3–22	1	2	0–6
Clinic Service						
Medicine	23	48	34–62	9	19	8–30
Surgery	25	52	38–66	3	6	0–13
Diagnosis Category						
Head	4	8	1–16	0	0	0
Abdomen	15	31	18–44	7	15	5–25
Repro	5	10	2–19	3	6	5–8
Limbs	16	33	20–47	1	2	0–6
Systemic	5	10	2–19	1	2	0–6
Healthy	3	6	0–13	0	0	0
Outcome						
Discharged	40	83	73–94	11	23	11–35
Died	1	2	0–6	1	2	0–6
Euthanised	7	15	5–25	0		0
Location Category						
General	21	44	30–58	3	6	0–13
Colic	9	19	8–30	3	6	0–13
Paediatric	8	17	6–27	3	6	0–13
Semi-isolation	3	6	0–13	2	4	0–10
Isolation	1	2	0–6	1	2	0–6
Outpatient	6	12	3–22	0	0	0
Pyrexia						
Yes	12	25	13–37	5	10	2–20
No	36	75	63–87	7	15	5–25
Diarrhoea						
Yes	6	12	3–22	3	6	0–13
No	42	88	78–97	9	19	8–30
Neutropaenia						
Yes	10	21	10–32	5	10	2–20
No	15	31	18–44	6	13	3–22
Not tested	23	48	34–62	1	2	0–6
Carrier Status (*n* = 12)						
Salmonellosis	-	-	-	2	17	0–10
Hospital-acquired	-	-	-	3	58	0–13
Carrier	-	-	-	7	25	5–25

**Table 2:** Distribution of *Salmonella* serotypes from positive patient and environmental samples submitted to the equine veterinary academic hospital between 30 October 2016 and 12 December 2016.

Serotype	Clinic Area	Stables	Patients	Total
	% (*n*/N)	% (*n*/N)	% (*n*/N)	% (*n*/N)
*S.* Typhimurium	84 (21/25)	72 (13/18)	42 (5/12)	71 (39/55)
*Salm* Poly OMD	-	16 (3/18)	17 (2/12)	9 (5/55)
*S.* Newport	8 (2/25)	-	-	4 (2/55)
*S.* Heidelberg	4 (1/25)	-	8 (1/12)	4 (2/55)
*Salm* II	-	6 (1/18)	8 (1/12)	4 (2/55)
*S.* Virchow	-	-	17 (2/12)	4 (2/55)
Rough-Untypeable	4 (1/25)	-	-	2 (1/55)
*S.* Schwarzengrund	-	6 (1/18)	-	2 (1/55)
*S.* Braenderup	-	-	8 (1/12)	2 (1/55)

## Data Availability

The original contributions presented in this study are included in the article/Supplementary Materials. Further inquiries can be directed to the corresponding author(s).

## Supplementary Materials

The following supporting information can be downloaded at: https://www.mdpi.com/article/10.3390/vetsci13040331/s1, Questionnaire S1 File: Public Health Questionnaire; Questionnaire S2 File: Biosecurity Questionnaire.

## Abbreviations

The following abbreviations are used in this manuscript:

SOP	Standard operating procedure
XLD	Xylose Lysine Deoxycholate
OR	Odds ratio
QAC	Quaternary ammonium compound
NaDCC	Sodium dichloroisocyanurate
CA	Citric acid
MPS	Potassium peroxymonosulfate
