# Supplementary material for: Epidemiology of a Salmonella Outbreak at a South African Equine Veterinary Academic Hospital Between October and December 2016
Source: Vet Sci. 2026 Mar 29;13(4):331. doi: 10.3390/vetsci13040331 (PMC13119749; doi:10.3390/vetsci13040331)
Supplement: Supplementary file 1 [file vetsci-13-00331-s001.zip › vetsci-4189081-supplementary.pdf]

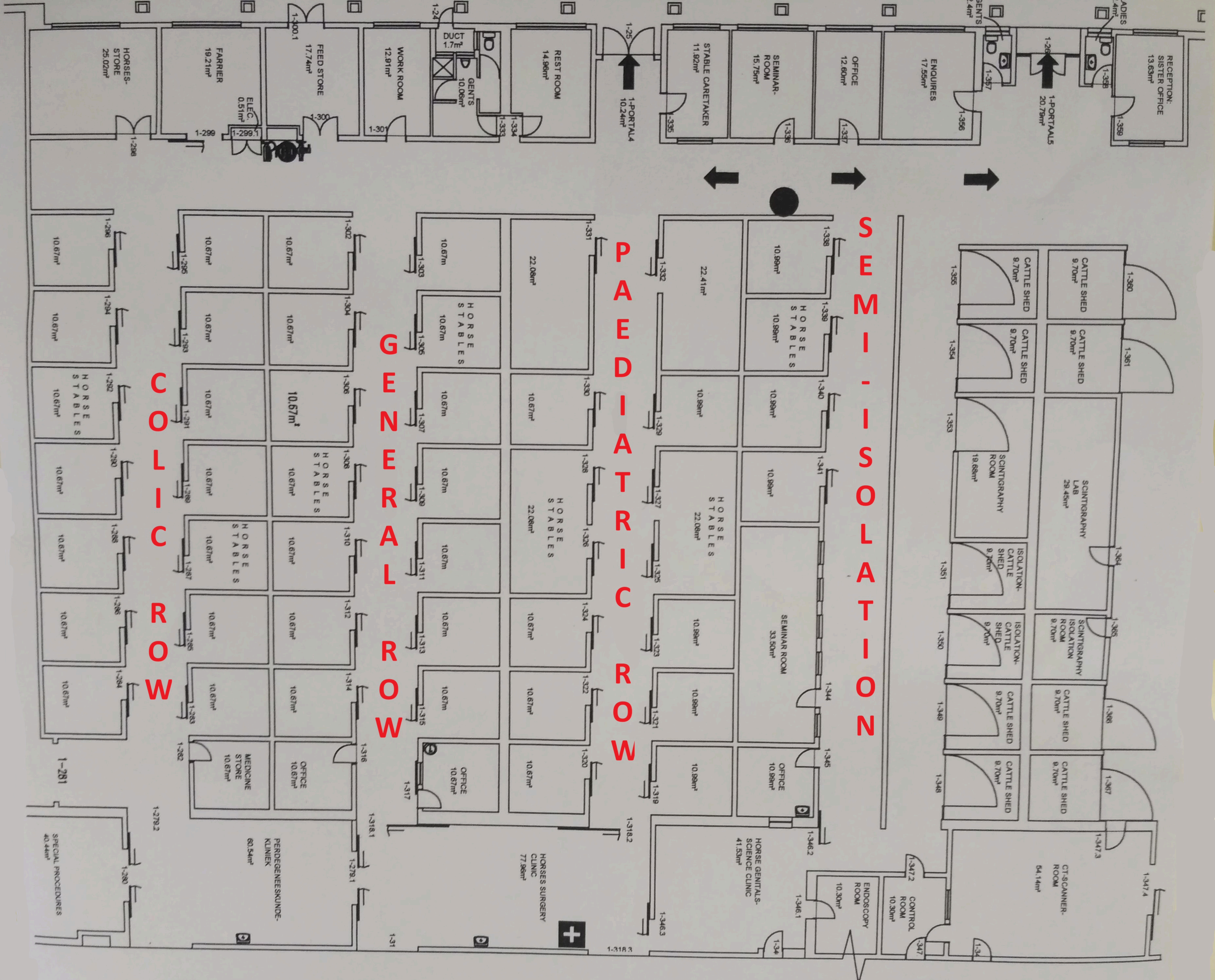

The padded downer stable and isolation stables are not depicted in this diagram. The downer stable is opposite the CT scanner and the isolation stables are located outside, approximately 100m from the main stable building

CONSENT FORM

Tahiyya (Tibz) Shaik and Nenene Qekwana
University of Pretoria, South Africa

Email: tibz.shaik@up.ac.za
nenene.qekwana@up.ac.za

You have been invited to take part in a research survey investigating the factors contributing to the *Salmonella* outbreak at the Onderstepoort Equine Clinic between October and December 2016 and the risk on public health. The survey will take approximately 3 minutes to complete. Taking part in this study is completely voluntary. If you choose to participate in this study, you can withdraw at any time. Your responses will be kept strictly confidential and anonymous. Any reports or publications that result from this research will be done at an aggregated level.

If you have questions or want a copy or summary of this study's results, you can contact us at the email address above.

Please feel free to print a copy of this consent page to keep for your records.

❍ I have read the above information and I agree to participate in this study

Name:_______________________________________

Sign:____

S1: Public Health Questionaire

Question 1: Were you present in the Equine Clinic between the 30th October 2016 and the 12th of December 2016? Please specify dates.

Question 2a: Were you sick during this time period?

Question 2b: Did you experience any of the following symptoms. Please tick.

- Pyrexia
- Nausea
- Vomiting
- Abdominal cramps
- Constipation
- Diarrhoea
- Other Please specify____

Question 2c: Did you consult a doctor?

Question 2d: What was your diagnosis?

Question 3: Would you be willing to share your medical records for this time period with us?

S2: Biosecurity Questionnaire

Question 1: How often was the clinic cleaned prior to the outbreak?

Each row has a SOP of how it should be cleaned and disinfected please use the hygiene program for your guidance attached below.

Low risk areas:

Stables: are mucked out on a daily basis and shavings are chucked out in the dumpster area for which is collected by the wastegroup
Passage: sweep only on Tue, Wed, Thurs, Sat, Sun and Mon and Thurs Blue-scrub and Foam after wards allow ½ exposure time

High risk areas:

Everyone involved in the care of the patient is obligated to wear new protective clothing (gloves, gowns, overshoes) for each patient: dispose them after use

Stables are mucked out on a daily basis and shavings are chucked out in the dumpster area for collection, if horse comes back positive while it's still in the hospital then the shavings are put in bags for incineration.

Passages Once a day after all the stables have been cleaned, Blue-scrub, rinse and sweep water away
Mon and Fri: Foam after blue-scrub allow ½ exposure time

Breezeway:

Mon and Fri: Blue-scrub then foam

Clinics:

Blue-scrub after use, if not Twice a week then foam

Feed store:

Sweep daily and mop with XD500 and disinfect with using F10SC

Workshop:

Sweep daily and mop with XD500 and disinfect with using F10SC

ICU Store room:

Sweep daily and mop with XD500 and disinfect with using F10SC

Mini-Pharmacy:

Sweep daily and mop with XD500 and disinfect with using F10SC

Western Passage incl Farrier room

Question 2: How often is the clinic cleaned after the outbreak?

Please refer to the hygiene program below

Question 3: What chemicals were used for cleaning prior to the outbreak?

Blue-scrub: 40ml of XD500 Detergent 10lt water (negative patient)
Green-scrub: 40ml XD500 with 300ml bleach in 10lt water (positive patient)
Foam: F10SC 1:250 (negative patient)
Bleach: 1:12(positive patient)

Question 4: What chemicals were used for cleaning during the outbreak?

Blue-scrub: 40ml of XD500 Detergent 10lt water (negative patient)
Green-scrub: 40ml XD500 with 300ml bleach in 10lt water (positive patient)
Foam: F10SC 1:110 (negative patient)
Bleach: 1:12(positive patient)

Question 5: What chemicals are being used after the outbreak?

Blue-scrub: 40ml of XD500 Detergent 10lt water (negative patient)
Green-scrub: 40ml XD500 with 300mlbleach in 10lt water (positive patient)
Foam: F10SC 1:250 (negative patient) 1:12(positive patient)

Question 6: Please explain the cleaning procedure prior to the outbreak

Refer to question 1

Question 7: Please explain the cleaning procedure during the outbreak?

Each row has a SOP of how it should be cleaned and disinfected
during the outbreak management decided to close down the clinic, which made it a lot easier for us to clean, Janet compiled a hygiene program for us to use as a guideline.

So for me to make sure the clinic gets cleaned at minimal time, me and the guys decided to clean the entire clinic including stables that were already cleaned, so we are sure we don’t miss anything.

For starters we used a WAP machine to get all the grease out, once that’s done, we then followed the hygiene program ratios. We cleaned out all the rooms e.g. feed store, workshop, pharmacy, office, student’s room.

Please refer to the documents I attached

Question 8: Please explain the cleaning procedure after the outbreak?

Please refer to the documents attached, so I have attached a normal and a salmonella program for reference

Question 9: Are different areas cleaned differently? Please elaborate if yes

Yes, refer to question 1

Question 10: What chemical was used in the foot baths prior to the outbreak?

Virkon

Question 11: What chemical was used in the foot baths after the outbreak?

F10SC

Question 12: What is your opinion of the previous cleaning regime?

I feel measurements are a bit low for a size of a stable for example, sometimes you get stables that are really dirty and you will need to add a bit more to get enough ‘foam’ to clean. I feel that it’s something we need to review.

Question 13: What is your opinion of the current cleaning regime?

The above mentioned, I still feel we need to adjust the measurements, and change our cleaning products every “agreed term”, I feel if we still continue to use the same products for more than 5 years some bacteria will become resistant, in my knowledge correct me if I am wrong.

Question 14: In your opinion what were the factors that contributed to the quick resolution of the outbreak?

Closing down of the clinic
Team work from the animal caretakers; we set out goals on how many areas needed to be cleaned in a day and it took us about 2-3 weeks to eradicate the problem and re-open again
Quick response from Bacteriology lab

Question 15: Do you have any further comments regarding the biosecurity in the equine clinic?

If everyone complies with the bio-security it will reduce the spread, we have a lot of visitors coming during the day and some don't step on the foot baths unless you see them.
There is no supervision of students during the night and I feel a lot of rules are bent during that time.
If people, mostly new students adheres to the bio-security that they are orientated on Mondays it can make a huge difference.
